# Cajal and the Conceptual Weakness of Neural Sciences

**DOI:** 10.3389/fnana.2015.00128

**Published:** 2015-09-30

**Authors:** José M. Delgado-García

**Affiliations:** Division of Neurosciences, Pablo de Olavide UniversitySeville, Spain

**Keywords:** Ramón y Cajal, neural sciences, neural functional laws and principles, neural plasticity and elasticity, higher brain functions

## Abstract

The experimental and conceptual contributions of Santiago Ramón y Cajal remain almost as fresh and valuable as when his original proposals were published more than a century ago—a rare example, contrasting with other related sciences. His basic concepts on the neuron as the main building block of the central nervous system, the dynamic polarization principle as a way to understand how neurons deal with ongoing active processes, and brain local structural arrangements as a result of the functional specialization of selected neural circuits are concepts still surviving in present research papers dealing with brain function during the performance of cognitive and/or behavioral activities. What is more, the central dogma of the Neuroscience of today, i.e., brain plasticity as the morpho-functional substrate of memory and learning processes, was already proposed and documented with notable insights by Ramón y Cajal. From this background, I will try to discuss in this chapter which new functional and structural concepts have been introduced in contemporary Neuroscience and how we will be able to construct a set of basic principles underlying brain functions for the twenty-first century.

## A Man of Laws and Principles

“The reason of the unreason with which my reason is afflicted so weakens my reason that with reason I murmur …”

*—Miguel de Cervantes, Don Quixote of La Mancha*

There are two main ways to increase the size and scope of a given science, and from that perspective Neuroscience is no exception. The easier one is to develop a new instrument (electron microscope, patch-clamp, functional magnetic resonance imaging) capable of providing original data impossible to obtain with existing devices. More difficult is to provide new conceptual and fruitful insights aimed at illuminating the core of the selected science, opening new approaches and pathways to advance it to still-unknown regions. In accordance with the title of this short review of Santiago Ramón y Cajal’s contributions to the development of modern Neuroscience, present-day neuroscientists are not particularly characterized by their ability to generate general principles capable of supporting and assimilating the large amount of experimental data collected during the past century (Delgado-García, 2006, 2011).

Besides his experimental contributions to the proper visualization and description of the cellular composition of the nervous tissue, Ramón y Cajal was a man of laws and principles, always ready to find and to propose the conceptual networks supporting and integrating his experimental findings. For example, Ramón y Cajal (1923) summarizes the four laws governing the morphology and connectivity of nerve cells as follows: (a) axons terminate in free ends; (b) axons lean against somas or dendrites; (c) somas and dendrites also participate in nerve impulses; and (d) those impulses are transmitted across those protoplasmic contacts. Making a comparison with the electrical instrumentation available in his time, Ramón y Cajal imagines that neural transmission takes place in the same way as current transmitted in the connections of electric components by a sort of induction, as in the coils of the same name.

Impressively, the first chapter of his *Textura del sistema nervioso* (1899) is full of principles relevant to the general design and evolution of the nervous systems of invertebrates and vertebrates. In the fifth chapter of that book, Ramón y Cajal describes another three laws fundamental in the organization of all nervous systems: (a) *law of economy of time*, by which neural pathways are as short as possible, adopting imaginative geometric solutions to this aim; (b) *law of economy of matter*, explaining why some axons are originated from somas or principal dendrites depending on their optimal location with relation to their projecting sites; and (c) *law of economy of space*, according to which neuronal somas and their protrusions are arranged in such a way as to avoid empty spaces around them. Finally, in chapter XLVIII of the above-mentioned book, Ramón y Cajal describes a series of functional postulates implied in the organization of cortical centers and pathways: (a) *unity of spatial and tonal perception*, referring to the cognitive need to generate a single and integrated perception corresponding to the whole set of stimuli present in the external world; (b) *concentric symmetries*, predicting in some ways the existence of cortical maps, mainly for two important sensory (visual and auditory) modalities; and (c) the already described laws of space and protoplasmic savings.

An additional law proposed by Ramón y Cajal has, like many other of his proposed conceptual generalizations, important functional implications: the law of the nervous avalanche or volley (Llinás, 2003). The conduction of nerve impulses in the form of volleys allows the simultaneous activation of numerous neural populations from a single sensory stimulus; among other functional advantages, this enables the generation of appropriate functional responses from neural sites located far away from the stimulated site.

## The Enigmatic Arrows in Ramón y Cajal’s Drawings

From my modest point of view, the three fundamental contributions of Ramón y Cajal to past and present Neuroscience are his exhaustive description of the morphology and connectivity of the nerve cells, the proposal of neurons as the building blocks of every brain, and the *principle of dynamic polarization*. In this section I will address my comments to that functional principle. In his *Recuerdos* (1923), Ramón y Cajal writes that this dynamic polarization principle was developed initially in 1891. In accordance with additional findings and critical comments, the principle was further developed in the so-called *theory of axipetal* (orthodromic)* polarization*.

**Figure 1:**
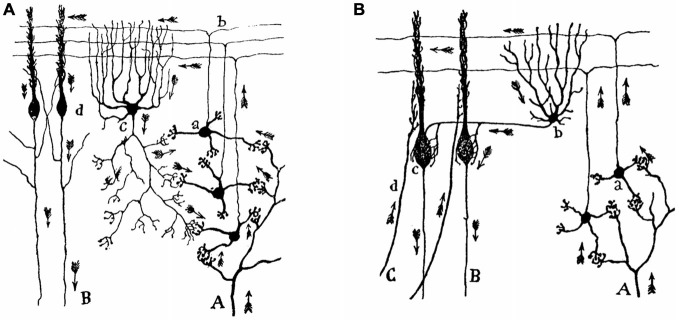
**Movement of the nerve impulse across the cerebellar circuit, according to Santiago Ramón y Cajal. (A)** An illustration of the direction and sense of the nerve impulse arriving along mossy fibers **(A)** at the granule cells (a) and from these, along the parallel fibers (b), at the Golgi (c) and the Purkinje (d) cells. The nerve impulse leaves the cerebral cortical circuit along Purkinje cell axons **(B)**. **(B)** Another view of the cerebellar cortical circuit including basket cells (b) and climbing fibers **(C)**. Taken from Ramón y Cajal, *Textura del sistema nervioso del hombre y de los animales* (1899–1904).

For Ramón y Cajal, the transmission of the nerve impulse takes place from the protoplasmic branches (i.e., the dendrites) to the neuronal body (i.e., the soma), and from this to the nervous expansion (i.e., to the axon). While dendrites and the soma represent a receptive device, the axon is the organ for transmission and distribution of neural messages. Ramón y Cajal developed his dynamic polarization principle from his detailed reconstructions of the (organized) cellular structure of the cerebellar cortex (Figure 1), but he successfully applied the same principle to many other neural circuits, such as, for example, to the interaction of sensory signals with motor commands in the cerebral cortex. He predicted that the centrifugal movement of voluntary motor commands transmitted across the two motor neurons (i.e., the projecting pyramidal cell and the motoneuron) to the skeletal muscles is originated in the dendrites of the pyramidal cells—that is, in the outer cortical layers, a cortical area receiving afferent sensitive, callosal, and other association fibers.

More than one hundred and twenty years after the seminal proposal by Ramón y Cajal, it is still difficult to explain how he was able to imagine the correct arrangement (direction and sense) of arrows included in his reconstructions of cortical and subcortical circuits. Indeed, to generate a dynamic principle for a dead and fixed tissue is (besides a contradiction) a genial interpretation of what one can see. Even now, it is difficult to imagine this orderly functional organization when looking through a microscope at a Golgi preparation of the cerebellar or the hippocampal cortices. Interestingly, with his functional proposal Ramón y Cajal not only was close to explaining the unidirectional character of chemical synapses, but also offered the background for the proper interpretation of many neural circuits from a functional point of view.

This linear and two-dimensional model of neuronal organization and functioning still has many followers and still underlies our current concepts of brain activities during complex functions such as learning and memory processes. Furthermore, Ramón y Cajal makes important suggestions about the functional role played by cortical short-axon neurons in cognitive, emotional, and related functions as opposed to those played by long-axon (projecting) neurons more related to sensory perception or to the execution of motor commands. The concept of reverberant circuits put forward by Lorente de Nó (1938) can be considered another attempt at advancing the functional consequences of the dynamic polarization principle and overturn this interpretation of the brain as a garden of intertwining paths. Today, people studying complex and distributed cerebral functions such as sleep and awareness (Hobson and Friston, 2012), or neuronal functioning during the actual acquisition of new motor and cognitive abilities (Delgado-García and Gruart, 2006; Gruart et al., 2015), have realized that these functional cerebral states require an interpretation different to (although not necessarily more complex than!) those proposed by Ramón y Cajal many years ago.

On the basis of the seminal studies of Hamburger and Levi-Montalcini (1949), as well as on those carried out by so many others (see Hamburger, 1980; Levi-Montalcini et al., 1996), it is now possible to propose a (new) *trophic polarization principle*. This principle indicates the dependence of neurons in the adult mammalian brain on molecular (?) signals generated by their target neurons, and makes reference to the maintenance of neuronal connections and the survival of interconnected neurons in the adult brain (Delgado-García and Gruart, 2004). In this way, whereas the concept of dynamic polarization suggests a flux of nervous information from dendrites and soma towards axon terminals, the concept of trophic polarization means an antidromic flux of information from the neuronal target, across the axon, toward the soma of the innervating neuron.

## Nuclei vs. Cortices

**Figure 2:**
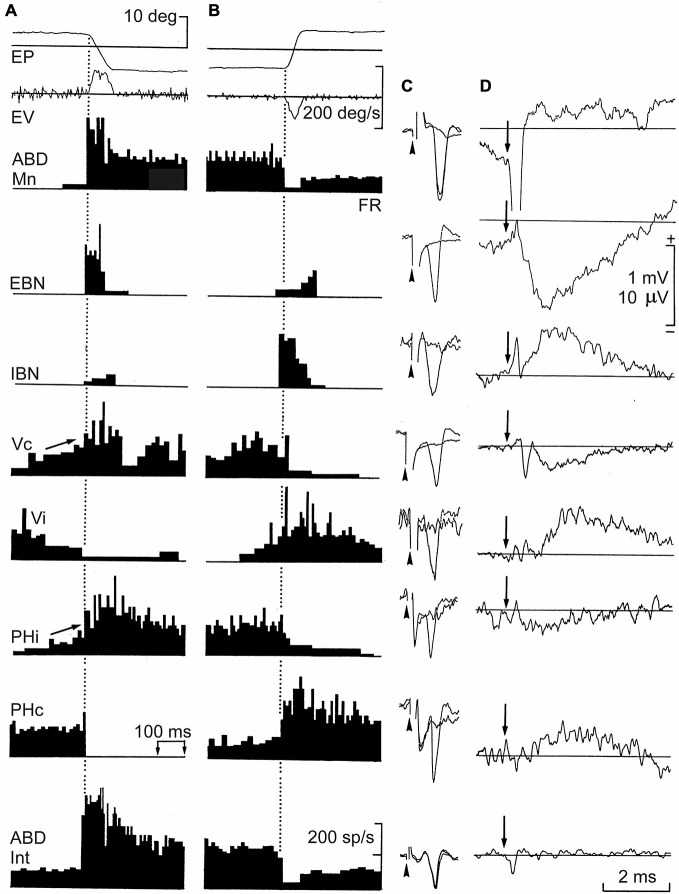
**Firing rate (FR), antidromic activation, and averaged field potentials induced by short-lead excitatory (EBN) and inhibitory burst neurons (IBN), and by ipsi- and contralateral vestibular (Vi, Vc) and prepositus (Phi, PHc) neurons projecting to the abducens (ABD) nucleus.** Recordings were carried out in alert cats. **(A,B)** Neuronal activity recorded from the illustrated neurons during spontaneous saccades in the on- and off-direction, respectively. **(C)** Antidromic all-or-none activation of recorded neurons by microstimulation of the ABD nucleus. Arrow-heads indicate the beginning of the stimulus. **(D)** Average of field potentials recorded in the ABD nucleus triggered (arrow) by these identified neurons. Abbreviations: EP and EV, horizontal eye position and velocity; FR. The discharge rate, antidromic identification from their projecting sites, and field potential induced by ABD motoneurons (Mns) and internuclear neurons (Int) are also shown. Taken with permission from Escudero and Delgado-García (1988).

In the very first chapter of his *Textura* (1989), Ramón y Cajal explains that the evolution of the nervous system leads to a functional specialization, which is latterly transformed into a morphological redefinition. As an expected result of the laws of time-, space-, and matter-saving it is logical that neurons carrying out similar functions pack together, forming nuclei. A coherent consequence of this principle is that the firing rate (FR) of a motoneuron located in a brainstem motor center can be representative of the firing activities of the other thousands of neurons located in the same nucleus (Delgado-García, 2011). The mammal abducens (ABD) nucleus is a good example of this proposal. This nucleus contains motoneurons (Mns) and internuclear interneurons (Int)—the first projecting to the extraocular lateral rectus muscle and the second projecting to the contralateral medial rectus subdivision of the oculomotor nucleus. Anyone following the evolution of these two groups of neurons in vertebrates will notice that in parallel with the progressive displacement of the two eyes to a frontal position, enabling a binocular vision, the two types of neuron approach progressively their somas in the pons as already determined in different species of mammals (Cabrera et al., 1993). Finally, these two types of neuron are perfectly intermingled in primates, constituting the ABD nucleus, where they share vestibular, reticular, and many other afferents to the nucleus (Figure 2; Escudero and Delgado-García, 1988).

Thus, as a functional consequence of the above contentions, we can generalize a principle by which neurons that are together in a nucleus fire under similar physiological rules. But, can we say the same for neurons packed together in layers? In this case, it is more than possible that the FR of a given Purkinje cell, or of a pyramidal neuron, is generated with a functional pattern different to the one shown by nearby neurons. When dealing with cortices, the functional consequences of this characteristic neuronal organization are not so evident. Nevertheless, the main contribution to advance the physiological rules of cortical arrangement was probably the concept of functional cortical column put forward initially by Mountcastle (1957), and later followed by many others (see Hubel and Wiesel, 1977).

Ramón y Cajal suggested that the appearance of the pyramidal cell enables the proper storage of sensory perceptions recollected from the external world in the form of ideas and volitions. In fact, in some of his early writings, Ramón y Cajal qualified the pyramidal cell as the *psychic neuron*, a concept that he seems later to have abandoned (López-Piñero, 2000; Goldman-Rakic, 2002). In any case, thanks to this specialized type of neuron, the perceived sensory events do not need to be immediately (and automatically) transformed into a motor response, but can be retained indefinitely by pyramidal cells, mainly by those located in association areas of the cerebral cortex. This information, stored in the form of memories in cortical cells, can be used later in the presence of new physical and/or social contingencies. For Ramón y Cajal, cortical association areas play an interconnection role between the more-specialized primary sensory and motor areas. It is possible that the peculiar organization of cortical circuits explains the difficulties for the proper identification of the functional codes of specific cortical sites—i.e., functional codes are modified moment by moment in accordance with the ongoing internal functional needs depending on the external constraints. According to Ramón y Cajal, the extension and structural complexity of the cortical gray layer are intimately related with the hierarchical psychological range of every vertebrate. More comments on this point are offered in the following section.

## Long Axons and Short Minds

**Figure 3:**
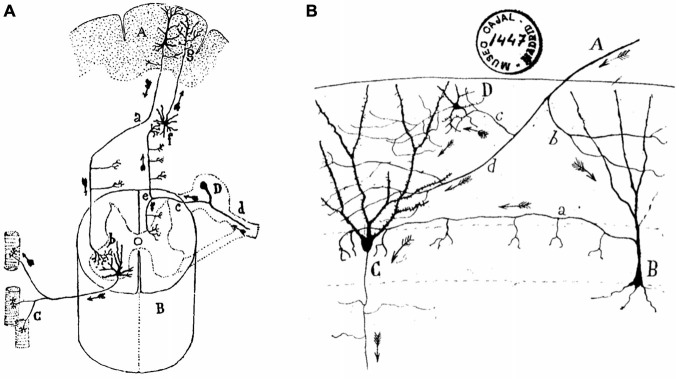
**Ramón y Cajal’s diagrams illustrating long-axon and short-axon neurons. (A)** Direction and sense of conscious motor commands and sensory signals in the nervous system. The diagram illustrates the cerebral cortical psychomotor region **(A)**, the spinal cord **(C)**, the muscle fibers **(C)**, and the spinal ganglion **(D)**. Motor commands descend by the pyramidal cell axons (a) which make contact with motoneuron dendrites (b). In turn, motoneuron axons terminate on the muscle fibers of their corresponding motor unit. Sensory signals arrive from peripheral receptors (d), and across the dorsal root (c) and the ascending tract (e) reach second-order neurons (f), which project to cerebral cortical neurons (g), where Ramón y Cajal assumes that they make contact with the protoplasmic branches (dendrites) of pyramidal cells. **(B)** An illustration of the twists and turns followed by axonal afferents to the dentate gyrus (DG) across short-axon neurons. **(A)** afferent fiber; **(B)**, short-axon neuron terminating around the somas of granule cells **(C)**; **(D)**, another type of short-axon neuron; b, c, d, different branches of the afferent fiber. Taken from Ramón y Cajal, Textura del sistema nervioso del hombre y de los animales (1899–1904).

Apparently, Ramón y Cajal did not like the distinction made by Golgi between sensory and motor neurons. In contrast, and as another example of his insightful generalizations, he divided nerve cells into those presenting short axons, branched and restricted inside the gray matter of the cortex, and those of long axons, constituting the white matter, bundles, and nerves (De Castro, 1981). Whereas it seemed clear for Ramón y Cajal that long-axon cells take care of rapidly sending sensory or motor messages far away in the peripheral and central nervous systems, he wondered about the reason for the presence of so many short-axon neurons interposed in, or collateral to the main neural pathways (Figure 3). Almost at the end of his *Recuerdos* (1923), Ramón y Cajal suggests that the functional excellence of the human brain is intimately related to the enormous abundance in number and in different structural displays of the so-called short-axon neurons. Indeed, short-axon neurons are mainly located in structures not immediately related to the generation of reflex responses, such as the cerebral cortex, the striatum, the thalamus, or the optic lobes. These short-axon neurons would act (in a still-unknown way) as peculiar stores for the psychic energy, being responsible for higher functions such as memory, ideation, and decision-making. In contrast, long-axon neurons would take care of the more bureaucratic role of carrying sensory information to the corresponding cortical sites, or motor commands to peripheral effectors.

Throughout his writings, Ramón y Cajal appears to be conscious of the extreme difficulties in proposing an architectonic and dynamic plan of the cerebral cortex able to explain mental functions. As he says, the cerebral cortex is like a forest in which many researchers have got lost. Indeed, he expects that future neuronal engineers will be the ones finding the proper pathways in this dense forest, an expectation not yet fulfilled. At least Ramón y Cajal realized that complex cognitive functions should be the result of the combined actions of a large number of commemorative cortical primary and secondary areas. Indeed, the cortex receives a huge amount of selective sensory information, allowing not only an appropriate internal representation of the external world, but also the internal design of appropriate motor responses. Years later, Llinás (1988) describes the same ideas in some more-contemporary words: “the mind is a computational state of the brain generated by the interaction between the external world and internal set of reference frames”. Never was knight so honored by his followers!

For his proposal of the putative roles of short-axon interneurons, Ramón y Cajal did not take into consideration the existence of inhibitory neurons, usually located in the circuits collateral to the main neuronal pathways that he drew with his habitual precision (Sotelo, 2003). Interestingly, some contemporaries of Ramón y Cajal were already aware of the presence of active inhibitory mechanisms in the brain of animals—as for example in the description by Pavlov (1927) of habituation as an active inhibitory process. Also, Freud (1895), in one of his early books (*Project for a scientific psychology*), proposes the concept of repression as an active inhibition of psychic energy, including a rather elementary scheme of brain circuitry: when this *psychic energy* is actively prevented from following a given neural pathway, it has to be redirected to other neural sites. In this way, the psychic energy is understood as a quantifiable entity. It is a pity that even today nobody has been able to put coefficients to this metaphoric form of energy, a fact contrasting with the remarks made in the following section.

## A Still-Unstated Principle

In my opinion, there is an important part of cerebral functions that can currently be understood with no major difficulties. Indeed, motor functions can be reasonably explained with the help of I. Newton’s second law (i.e., acceleration is produced when a force acts on a mass). This proposal was fruitfully developed in the past 50 years by Robinson (1981) and his followers, up to convincingly explaining the intrinsic neural organization of the extraocular motor system (Delgado-García, 2000). From an evolutionary point of view, it can be proposed that brains were originated by the need for movement at a certain speed in a three-dimensional world in which vertebrate bodies have to move against gravitational forces, and also have to deal with the viscous and elastic components of the external physical elements (see Llinás, 1988).

It is remarkable that all written contributions from Ramón y Cajal are always illuminated by the dynamic contributions of his cellular and histological findings (Sotelo, 2003). In this regard, and in the light of the many different morphological types of neuron described in his *Textura* (1899–1904), how is it possible that neither Ramón y Cajal nor any of his contemporary colleagues raised the issue of this huge morphological diversity? What is that for? For example, Purkinje cells are quite different from the other four (or five) types of neuron present in the cerebellar cortex. In turn, those cortical cerebellar cells are noticeably different in shape and dimension from those located in the cerebral cortex, in the thalamus, or in the brainstem. All of us can readily agree that every neuronal type exhibits specific physiognomic profiles (De Castro, 1981), but what about their functional peculiarities? What about their physiology? Today, we can describe without much difficulty the *physiology* of the motoneuron: a specialized type of neural cell encoding the current length of the innervated muscle and the speed with which this length is modified. Perhaps we can also explain the physiology of retinal rods and cones as their capability to transduce a given band of the electromagnetic spectrum into biopotentials. What is not so easy is to offer similar synthetic functional descriptions for most central nervous system cells. What could be the physiology of inferior olive cells, of reticular, thalamic or pyramidal neurons, and even of the most complex known neuron, the Purkinje cell? More experimental approaches, ranging from immunohistochemistry to patch-clamp and from the precise determination of expressed constitutive and functional proteins to the presence of selective membrane receptors and related molecular elements, do not necessarily contribute to a better understanding of the functional reason why a given neuronal type needs to be right where it appears.

**Figure 4:**
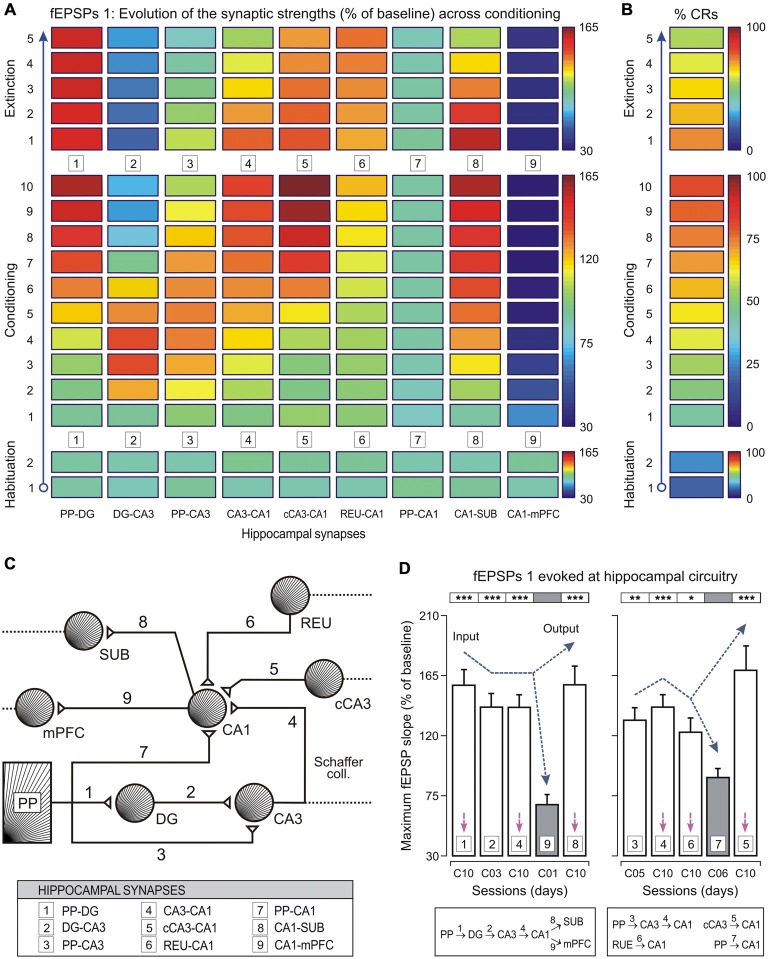
**Changes in strength of different hippocampal (and related) synapses during classical eyeblink conditioning of behaving mice. (A,B)** Evolution of field excitatory post-synaptic potential (fEPSP) slopes (as % of baseline, see **(A)**) collected at 9 synapses, and percentage of conditioned responses (% CRs, see **(B)**) across the successive training sessions. Selected synapses were: 1, perforant pathway (PP)–DG; 2, DG–CA3; 3, PP–CA3; 4, CA3–CA1; 5, contralateral CA3 (cCA3)–CA1; 6, thalamic reuniens nucleus (REU)–CA1; 7, PP–CA1; 8, CA1–subiculum (SUB); and 9, CA1–medial prefrontal cortex (mPFC). Note that some synapses increased in learning-dependent strength across training (PP–DG, CA3–CA1, cCA3–CA1, REU–CA1, and CA1–SUB), whilst for others their strengths increased at the beginning of the conditioning sessions and decreased at the end (DG–CA3, and PP–CA3). Finally, some of them did not present any significant change (PP–CA1), or decreased across training (CA1–mPFC). See code color bars to the right of **(A,B)**. **(C)** A diagram of hippocampal synapses included in this study, indicating their main input and output connections. **(D)** Mean values of maximum synaptic strength across learning for the 9 synapses. According to the statistical analyses, 4 synapses (1, 2, 4, and 8) presented maximum synaptic strengths significantly different from those evoked by synapse 9 (left histogram). Note the potentiation of synapse 8 in contrast to the inhibition of synapse 9 during the acquisition process. A similar analysis (right histogram) showed that synapses 3, 4, 6, and 5 presented significantly different maximum synaptic strengths from those shown by synapse 7. However, synapses 1, 4, 5, and 8 presented similar synaptic strengths at the asymptotic level (session C10, see magenta arrows) of the acquisition process. Error bars represent SEM. Taken with permission from Gruart et al. (2015).

In any case, it seems convenient to start the outline of a new functional principle that we could initially refer to as the *transformation principle*. It is clear that the different ionic conductances present in the available neuronal types confer on them quite diverse functional properties. The different neural types across a neural pathway (i.e., visual, auditory, motor, etc.) were classically considered passive relay points in the transmission of sensory percepts or motor commands. However, it seems reasonable to propose that every neural type present in a given pathway or circuit plays a specific and unique role. This uniqueness is dependent on their electrogenic and secretory properties, at the same time that the latter are dependent on the ionic conductances present in the plasmatic membrane and/or on the types of protein expressed inside the neuron. In this way, the nervous system cannot be considered a huge set of an enormous amount of neural elements capable of detecting sensory signals and of switching them into motor commands, but better as a distributed structure able to *transform* the incoming information from sensory receptors in an autonomous and highly organized internal world, not necessarily dependent on the external milieu. This internal milieu generated by the activity of hundreds of different types of neuron is capable of producing original decisions not necessarily contingent on external demands. It should be expected that a different functional state would correspond to each of the many different functional capabilities of the set of different neuronal types composing a brain (Figure 4). In accordance, each neuronal type described by Ramón y Cajal must have its own physiology. We need only to identify them.

## The Game is Between Plastics and Elastics

Were the brain as plastic as many contemporary neuroscientists seem to think, it would not be necessary to revisit the *Textura* (1899–1904), because neurons illustrated in the text would no longer exist. Nevertheless, Ramón y Cajal (1905) might be included in the *plastic* group—for example when he proposes that interneuronal connectivity, besides its hereditary origins, is susceptible to being influenced and modified during youthful years by education and habits. In fact, Ramón y Cajal was certainly influenced by the proposals of Tanzi (1983) and many others, regarding the motility of dendritic protrusions, including their spines, and that of axon terminals. Ramón y Cajal suggests that such motility might be dependent on the use of the involved neuronal connections, a fact obviously related with the psychomotor activities (such as sleep, memory, and thinking) of the subject. These thought-provoking proposals still survive among contemporary neuroscientists (Kandel, 1998, 1999; Albright et al., 2000; Yuste and Bonhoeffer, 2001; Sotelo, 2003). In consequence, either sleep processes (in Ramón y Cajal’s time) or learning and memory phenomena (in ours) are explained not on the basis of the intrinsic properties of neuronal circuitry, but on its capability of being modified. However, A. Von Kölliker (a contemporary of Ramón y Cajal) raised some concerns; he realized that while psychic processes are fairly stable in the same subject, amoeboid changes in neuronal connections are continuous and disorganized, mostly related to nutritional and thermal phenomena. Indeed, neurons and glial cells are capable of a certain motility (Ramón y Cajal, 1899–1904; Bonhoeffer and Yuste, 2002); the point is how to establish proper causal relationships between conformational changes in neuronal connectivity and adaptive modifications in the behavior and mental status of the subject. Ramón y Cajal envisages that some changes in neuronal connectivity (lazy connections, partial disconnections of commemorative systems) could be the substrate for selective neural pathologies. In other words, neural plastic changes will not always be beneficial for the supporting individual!

Assuming that plastic changes are those that remain for long periods of time, the concept of neural plasticity is not applicable to the regeneration of nervous tissue after a lesion. In such case, it would be better to talk of *elasticity*—i.e., a mechanism allowing the return to the preceding state. Peripheral nerve regrowth after a section is a well-known example of this notion. Another example is the retraction effect of both tetanus and botulinum neurotoxins on presynaptic axons terminating on the infected motoneuron (Pastor et al., 1997). Once the neurotoxic effects are over, all of the presynaptic terminals (a few thousand for a motoneuron!) return to the postsynaptic membrane in a similar proportion and with a similar functionality, allowing the complete recovery of the motoneuron physiology (Pastor et al., 1997; Delgado-García and Gruart, 2004). Thus, the nervous system is not only capable of modifying its intrinsic connectivity in a sustained way (for learning to occur), but also of returning to its previous state after some types of neural lesion (for regenerative processes).

In accordance with the law of the morphological progress (Ramón y Cajal, 1923; De Castro, 1981), neurons would add new appendages to their terminals and would increase their connections with other neural cells as a result of the increasing functional adaptability. For Ramón y Cajal (at the Rome Meeting, 1894), these plastic phenomena are more frequent and abundant in the cerebral cortex, in contrast to more-stable centers (evolution-ankylosed systems) such as the brainstem and the spinal cord. From an evolutionary perspective, it is reasonable to think that very old neural systems with a well-defined function are less susceptible to modification than those appearing more recently. An example of the former is the vestibular system, whose more-recent adaptive changes took place a hundred million years ago (Delgado-García, 2000), while a good example of the latter is the association cortex, very susceptible to modification precisely because of its functional indefinition (Gould, 1980).

To finish this section, and taking advantage of Kölliker’s suggestions (see above), we should keep in mind that the brain also has a tendency to compensate for unwanted changes (as in the early stages of many neurodegenerative diseases). These *stability* functions are probably more evident in the older neural systems. For example, extraocular Mns can easily compensate for the experimental removal of a considerable percentage of presynaptic afferents, a phenomenon less readily observed in cortical pyramidal cells (Delgado-García, 2011).

## Ready to Depart

Although from time to time we are presented (Bullock, 1959; Shepherd, 1972; Bennett, 2002; Bullock et al., 2005; Guillery, 2005) with (rather timid) criticisms of Ramón y Cajal’s neuronal doctrine, the truth is that we still lack a really new conceptual framework of brain structure and functions capable of surpassing the preceding proposal. I should expect a big jump from Ramón y Cajal’s arrows in a two-dimensional space to a three- or fourth-dimensional brain analyzed during its functional states: sleeping, dreaming, thinking, learning, and remembering. Apart from some recent attempts (for example, results collected using functional magnetic resonance imaging and related techniques; Fox and Raichle, 2007; Yao et al., 2015), the path followed was the opposite, and experimental neuroscientists mostly preferred to move toward the inside of the neuron (Kandel, 1998, 1999; Albright et al., 2000; Changeux, 2001) in search of the molecules that make possible learning, social interactions, or attentive phenomena. In the words of Santiago Ramón y Cajal, it seems that we are becoming followers of the religion of the small (trivial?). Perhaps in the reasonable curiosity to find at the lower integration level the explanation for a given functional process, we are at risk of forgetting the search for the origin of emergent properties emanating from the brain *in vivo*.

**Figure 5:**
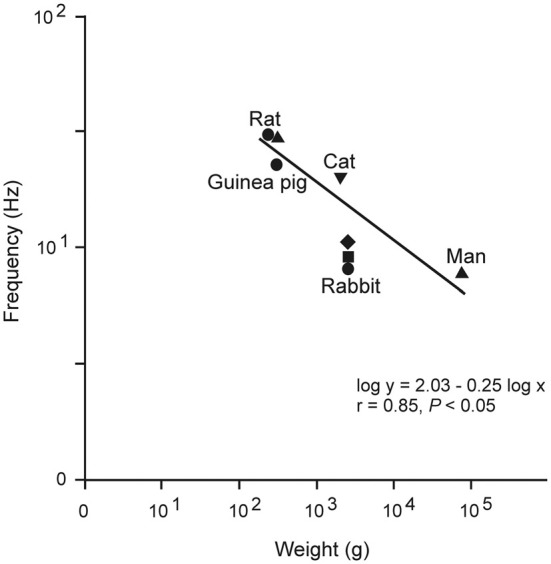
**Relationship between mean body weight and the dominant frequency of eyelid responses for different species.** Plot illustrates data obtained from rats, guinea pigs, cats, rabbits, and humans. These data suggest that lid biomechanics is tuned to the weight and viscoelastic properties of the lid. Note that the slope of this relationship (−0.25) is the same as that characterizing heart rate and body mass (in kg) in mammals. Taken with permission from Gruart et al. (2000).

Figure 5 illustrates an example of emergent properties rather difficult to identify by a study of the intracellular properties and compositions of the involved neural elements. In contrast, the emergent resonant oscillatory property of the eyelids was obtained from physiological recordings carried out, for comparative purposes, in different species of mammals (Gruart et al., 2000).

Apart from the above considerations, at the time of explaining brain properties we are too prone to use metaphors originated from our scientific and cultural surroundings. For example, Descartes imagined brain function in the light of the pneumatic systems then available in the gardens at Versailles. Mendeleev attempted to explain human behavior following rules obtained from his famous periodic table. Pavlov, Freud, and Skinner tried to describe both behavior and mental states in accordance with their respective discoveries. Even Ramón y Cajal assumed that interneuronal communication took place similarly to the electrical devices (accumulators, induction coils) available in his time, and compared neuronal connectivity to the emerging telephonic system. We are still in a similar tessitura: “if we ever do find some powerful generalization that applies to brains in particular then it will probably not be about brains as a piece of biology but about brains as computing entities” (Guillery, 2005). Although it is possible that in the near future our industrial products look as similar to us as sons are similar to their fathers, it is evident that this metaphoric approach (in the absence of appropriate coefficients and quantifications) seems not very productive in the mid or long term.

Have contemporary Neuroscience feet of clay? Parodying the title of Ramón y Cajal’s major book, the brain is a text from which we know precisely its texture (thanks to his contributions, as well as to those of many other neuroscientists), but we are still incapable of understanding its semantics.

## Conflict of Interest Statement

The author declares that the research was conducted in the absence of any commercial or financial relationships that could be construed as a potential conflict of interest.
